# The impact of a pre-loaded multi-ingredient performance supplement on muscle soreness and performance following downhill running

**DOI:** 10.1186/s12970-014-0063-6

**Published:** 2015-01-21

**Authors:** Michael J Ormsbee, Emery G Ward, Christopher W Bach, Paul J Arciero, Andrew J McKune, Lynn B Panton

**Affiliations:** Department of Nutrition, Food and Exercise Sciences, Florida State University, Tallahassee, FL USA; Institute of Sports Sciences and Medicine, Florida State University, Tallahassee, FL USA; Discipline of Biokinetics, Exercise and Leisure Sciences, University of KwaZulu-Natal, Durban, South Africa; Human Nutrition and Metabolism Laboratory, Skidmore College, Saratoga Springs, NY USA

**Keywords:** Eccentric exercise, Muscle damage, Recovery

## Abstract

The effects of multi-ingredient performance supplements (MIPS) on perceived soreness, strength, flexibility and vertical jump performance following eccentric exercise are unknown. The purpose of this study was to determine the impact of MIPS (NO-Shotgun®) pre-loaded 4 weeks prior to a single bout of downhill running (DHR) on muscle soreness and performance. Trained male runners (*n* = 20) were stratified by VO_2max_, strength, and lean mass into two groups; MIPS (*n* = 10) ingested one serving daily of NO-Shotgun® for 28 days and 30 min prior to all post-testing visits, Control (CON; *n* = 10) consumed an isocaloric maltodextrin placebo in an identical manner as MIPS. Perceived soreness and performance measurements (strength, flexibility, and jump height) were tested on 6 occasions; 28 days prior to DHR, immediately before DHR (PRE), immediately post (POST) DHR, 24, 48, and 72 hr post-DHR. Perceived soreness significantly increased (p < 0.05) post DHR compared to PRE at all time-points, with no difference between groups. Creatine kinase (CK) and lactate dehydrogenase (LDH) increased over time (p < 0.001) with no group x time interactions (p = 0.236 and p = 0.535, respectively). Significant time effects were measured for strength (p = 0.001), flexibility (p = 0.025) and vertical jump (p < 0.001). There were no group x time interactions for any performance measurements. Consumption of MIPS for 4 weeks prior to a single bout of DHR did not affect perceived soreness, muscle damage, strength, flexibility, or jump performance compared to an isocaloric placebo in trained male runners following a single bout of DHR.

## Background

Exercise induced muscle damage (EIMD) from unaccustomed bouts of eccentric or strenuous exercise has been well documented [1-4]. EIMD promotes an unbalanced ratio of protein breakdown to protein synthesis and is associated with increased muscle soreness, decrements in strength, and impaired muscle function for several days post-exercise [5]. Muscle damage induced by strenuous or unaccustomed downhill running (DHR) initiates biochemical and skeletal muscle morphology changes indicated by localized edema, increased circulating inflammatory markers (cytokines and leukocytes), and increases in indirect markers of muscle damage (creatine kinase (CK), lactate dehydrogenase (LDH)) [3,6].

In parallel with elevated markers of tissue damage following eccentric exercise are decrements in performance measures, including muscular power, maximal contractile force (eccentric, concentric, isometric), and endurance performance [2,7,8]. Previous studies have demonstrated a positive impact on recovery from EIMD through supplementation with whey protein isolate [5], branched-chain amino acids (BCAAs: leucine, isoleucine, and valine) [9,10], leucine only [2], creatine [11,12], and caffeine [13,14]. However, little is known about the effects of multi-ingredient performance supplements (MIPS) [i.e. common MIPS components: protein, BCAAs, creatine, beta-alanine, caffeine, and l-arginine] on the attenuation of muscle damage and repair after EIMD from DHR. Multiple studies have demonstrated beneficial effects following an identical 28-day, pre-exercise supplementation protocol [15-17]. While the improved strength and body composition variables from MIPS supplementation in these studies are promising, a resistance training protocol was used. However, the significantly increased muscle mass and strength gains with MIPS supplementation suggests the MIPS may have elicited improvements via specific ingredients included in the MIPS. Specifically, increased muscle protein synthesis and recovery (i.e. protein, BCAAs, leucine), hydrogen ion buffering (i.e. beta-alanine), ATP resynthesis (i.e. creatine), and/or improved CNS stimulation (i.e. caffeine) are plausible mechanisms of action.

While the aforementioned pre-exercise MIPS supplementation studies are promising [15-17], due to the use of a resistance training protocol, assumptions must be made to draw conclusions about the effects of MIPS on the attenuation of EIMD following a DHR in an endurance population. Therefore, the purpose of this study was to determine the impact of MIPS, pre-loaded 4 weeks prior to- and for 72 hr following a single bout of DHR on muscle damage, soreness, and performance during recovery. We hypothesized that this supplementation protocol would decrease ratings of perceived soreness, improve biochemical markers of muscle damage (i.e. CK and LDH), improve muscle performance of isokinetic and isometric strength, range of motion (ROM), and jump performance greater than an isocaloric placebo in endurance-trained male runners for up to 72 hr post-DHR.

## Methods

### Participants

Twenty healthy, male endurance-trained runners (maximal oxygen uptake (VO_2max_) ≥ 55 ml/kg/min and an average 20 miles/week of running), ages 18–50 years were recruited to participate in this study (Table 1). Participants were excluded if they had existing diseases (e.g. cardiovascular disease), musculoskeletal disorders, any history of leg injury or any other medical condition that would be exacerbated by a single bout of DHR, or the regular use of any anti-inflammatory drugs. Participants who were consuming any other dietary or ergogenic supplements were instructed to immediately stop consumption and complete a 4-week washout period before participating in the study (one participant reported use of a generic over-the-counter multivitamin supplement, no other supplement use was reported). All procedures involving human subjects were approved by the Florida State University Human Subjects Institutional Review Board, and written informed consent was obtained prior to participation. Throughout the study, all participants maintained their habitual diet, and were given 24-hr dietary food logs to record their meals the day prior to baseline testing and asked to replicate these meals before all other laboratory visits. Participants ate their last meal at least 6 hr prior to testing and consumed a commercial Chocolate Chip Clif Bar® (240 kcals) 3 hr prior to testing and at least 3 hr from their last meal.

**Table 1:** **Participant characteristics (** ***N*** **= 20)**

	**MIPS (** ***n*** **= 10)**	**CON (** ***n*** **= 10)**
Age (yrs)	24 ± 5	30 ± 10
Height (cm)	178.3 ± 5.6	181.9 ± 7.5
Body Mass (kg)	72.4 ± 8.3	74.4 ± 6.3
Body Fat (%)	9.2 ± 2.6	10.3 ± 3.1
VO_2max_ (ml/kg/min)	61.7 ± 5.3	61.4 ± 4.9

Values are expressed as mean ± SD.

MIPS: Multi-Ingredient Performance Supplement; CON: Control Carbohydrate Placebo; VO_2max_: Maximal Oxygen Uptake.

### Treatment

The study was a placebo-controlled, double blind protocol with two groups. After baseline testing participants were stratified by VO_2max,_ isometric voluntary contraction strength (60°), and lean mass and assigned to either MIPS (*n* = 10) or CON (*n* = 10). MIPS consumed one serving (21 g) of NO-Shotgun® (~72 kcals; 18 g protein; 9.7 g protein hydrolysate matrix including BCAAs; 8.06 g muscle volumizing and power/speed/strength matrix that includes multiple forms of creatine and beta alanine; 376 mg of Redline®energy matrix including caffeine; Vital Pharmaceuticals, Inc., Davie, FL) once per day for 4 weeks prior to the DHR and 30 min prior to all post-testing visits (3 days) with 355 - 473 ml of water. CON consumed one serving (21 g) of an isocaloric, flavor-matched placebo beverage (maltodextrin) once per day for 4 weeks prior to the DHR, as well as 30 min prior to all post-testing visits. All participants received their supplement in identical commercially labeled containers after baseline testing. Participants consumed either MIPS or CON 30 min prior to exercise on all exercise training days, or first thing in the morning on non-training days. Empty containers were collected to verify compliance from all participants.

### Experimental overview

**Figure 1:**
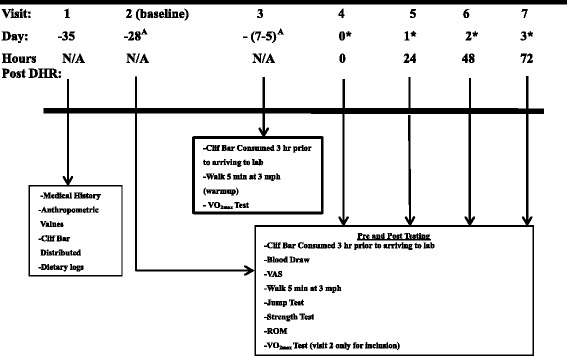
**Study timeline.** VAS: Visual Analog Scale, VO_2max_: Maximal Oxygen Uptake, ROM: Range of Motion. ^A^Multi-Ingredient Performance Supplement (MIPS) or Control (CON) consumed once daily from Visit 2 up to 1 day prior to Visit 4 (30 min prior to exercise on training days and immediately upon waking on non-training days); *MIPS or CON consumed 30 min prior to visits 4, 5, 6, and 7.

Participants reported to the laboratory on 7 different occasions (Figure 1). The first visit included a review of the informed consent document, distribution of the Chocolate Chip Clif Bar®, as well as the measurement of anthropometric values. The second laboratory visit was no later than 1 week from the first visit and was used as the baseline/inclusion testing for participation in the study, including VO_2max_ determination. Subsequent to performance testing on this visit, an incremental treadmill running protocol was employed to determine VO_2max_ using a motor driven treadmill (Woodway®, Waukesha, WI, USA). Expired air was measured breath-by-breath by indirect calorimetry using a metabolic measurement system (Parvomedics Truemax® 2400, Consentius Technologies, Sandy, UT, USA). Prior to the beginning of each test, the gas analyzer was calibrated using ambient air and a gas of a known composition containing 20.9% O_2_ and 4% CO_2_. The turbine flowmeter was calibrated using a 3-L syringe (Hans Rudolph, Inc., Kansas City, MO, USA).The participant began by walking for 5 min at 3 mph then the speed was increased to 5.5 mph at a 0% grade for 2 min (stage 1). Upon completion of the first stage the speed was increased 1 mph every 2 min until the participant reached the speed of 9.5 mph (stage 5). After the completion of stage 5 the participant maintained a running speed of 9.5 mph and the grade was increased by 2% every 2 min until the participant reached volitional fatigue. Heart rate was monitored using a heart rate monitor (Polar™, Lake Success, NY, USA). Ratings of perceived exertion (RPE) and heart rate were recorded at the end of each stage, as well as when participants reached volitional exhaustion, or requested to stop the test. The test was considered to be a maximal test under 2 conditions: 1) If the participants’ VO_2_ reached a plateau for more than 1 stage (2 min intervals) while intensity continued to increase or 2) Participants exhibited 2 of the following secondary criteria: RPE ≥ 19, respiratory exchange ratio (RER) ≥ 1.1, or a heart rate within 10 beats/min of the theoretical age predicted maximum heart rate (220 – age). The researchers provided verbal encouragement during the test to ensure maximal effort.

Upon completion of baseline testing the participants were given instructions on supplementation and instructed to maintain their normal dietary and training patterns for the next 28 days during supplementation. Approximately 3 weeks from baseline testing participants returned to the laboratory under the same pretesting conditions as baseline to perform a VO_2max_ test to determine their intensity (75% of VO_2max_) during the DHR.

A 10 ml venous blood sample was collected and perceived muscle soreness, jump height, isokinetic and isometric strength, and ROM were measured, respectively, during 5 different laboratory visits: baseline (28 days before DHR), immediately before the DHR (PRE), immediately after the DHR (POST), and 24, 48, and 72 hr post-DHR. After the blood draw and muscle soreness was measured, participants walked for 5 min at 3 mph on a motorized treadmill to warm-up prior to any performance measurements (the DHR was only performed during visit 4; See Figure 1). Prior to these visits participants consumed their commercial Chocolate Chip Clif Bar® (given during visit 1) 3 hr before testing and replicated their 24 hr dietary food logs. Participants were asked to refrain from physical activity 48 hr and caffeine, alcohol, and pain medication (i.e. ibuprofen) 24 hr prior to all laboratory testing visits.

### Measures

Muscle soreness was measured via a visual analog scale (0–100 mm). Participants rated their level of soreness of the rectus femoris, vastus medialis, vastus lateralis, biceps femoris, gastrocnemius, and gluteus maximus on the right leg by drawing an intersecting line across a continuum line extending from 0 mm (0 = no soreness) to 100 mm (100 = extreme soreness). To help quantify pain, an algometer (Force Ten™ Wagner Instruments, Greenwich, CT, USA) was used with an application of 4 kg of pressure. The midline of the muscle was determined using a tape measure and the algometer was placed at this point with the application of the 4 kg of pressure.

Jump height was assessed using a Vertec vertical jump system (Perform Better, Cranston, RI), recording the best of 5 attempts. The squat jump test was used to assess dynamic explosive force production of the leg extensors. With no restriction on knee angle during the eccentric phase of the knee flexion, participants were instructed to bend down with their knees shoulder width apart and explosively jump, reaching their hand as high as possible during the peak height of the jump to shift the tabs at the top of the Vertec bar. Participants completed 5 jumps with 30 seconds of rest between each jump with the highest measure recorded for analysis.

Isokinetic (5 repetitions, 180°/sec unilateral knee/extension flexion) and isometric (3 repetitions/5 second rest, 60° isometric knee extension/flexion) strength of the dominant leg were determined using the Biodex System 3 (Biodex Medical Systems, Shirley, New York) exercise dynamometer. Each participant was placed in the upright-seated position in the Biodex system, and the seat height and position were adjusted in order to align the instrument’s axis of rotation with the participant’s knee. Once positioned correctly and secured, ROM was determined along with the weight of the limb. The dominant leg was used for all Biodex testing. Participants were instructed to cross their arms over their chests without holding the restraints or handles. The first test was an isokinetic 180°/sec unilateral knee/extension flexion. Five repetitions of consecutive maximal extension and flexion concentric contractions were performed. Sixty seconds following the isokinetic test, a 60° isometric knee extension/flexion test was performed. The test involved 3 alternating maximal extension and flexion exertions against an immovable arm, with 5 second rest periods between exertions. Continuous verbal encouragement was provided by the research team throughout the duration of all tests. Participants were allowed to view the data reporting screen and all values as they were recorded. The peak torque of the isokinetic and isometric tests for the quadriceps and hamstrings were recorded for later analysis.

Range of motion (ROM) was measured by sit-and-reach assessment using standard testing procedures [18] (Figure Finder Flex Tester® box; Novel Products, Inc., Rockton, IL, USA). The best of 3 trials was recorded as the final value used for statistical analysis.

Height and weight were measured via the use of a wall-mounted SECA 216 stadiometer and a digital scale (SECA, Hamburg, Germany), respectively. All measurements were taken without shoes and wearing minimal clothing (e.g. running shorts). Body composition was measured non-invasively using the sum of 3 skinfold measurements (chest, abdomen, and thigh), all taken by the same technician, that were used to calculate body fat percentage [19].

Blood (10 mL) was collected via venipuncture of the antecubital vein at baseline testing, PRE, POST, 24, 48, and 72 hr after the DHR. Following the blood collection, serum (BD Vacutainer, no coagulant) was allowed to clot and centrifuged at 3500 RPM at 4°C for 15 min (Sorvall ST16R Multispeed Centrifuge; Thermo Electron Corporation, Needham Heights, Massachusetts). Aliquots (300 μL) were then transferred into microtubes and immediately frozen at −80°C for later batch analysis. Serum CK activity and LDH were analyzed in duplicate using commercially labeled assay kits (BioAssay Systems, EnzyChrom).

### Downhill running protocol

Prior to the DHR during visit 4, participants began by warming up for 5 min walking on a level grade at 3 mph. The treadmill grade was then lowered to -5% grade. Participants ran continuously for 60 min at a speed eliciting 75% of their VO_2max_ on a level grade (determined during visit 3) [18]. Heart rate and RPE were monitored continuously and recorded every 10 min during the DHR (data not shown).

### Statistical analysis

Sample size was determined using the data from the study by Hoffman et al. [8]. Using an alpha level of 0.05 and power of 80% an effect size of 0.88 was determined. Therefore, a total of 10 participants per group were needed. Participant characteristics and baseline data between groups were analyzed using one-way analysis of variance (ANOVA). Baseline data were also compared to PRE data (after 4 weeks of MIPS or CON) to see if supplementation affected measured parameters by using a 2×2 repeated measures ANOVA. The DHR data were analyzed using a 2 × 5 (group: MIPS or CON × time: PRE, POST, 24, 48, 72 hr) ANOVA with repeated measures. A LSD post-hoc test was used to examine pairwise differences if there were significant main effects. Greenhouse-Geisser analysis was used if the Mauchly’s Test of Sphericity was violated. Significance was set at *p* < 0.05. SPSS version 21.0 (SPSS Inc, Cary, NC) was used for statistical analyses. Data are presented as means ± standard deviations.

## Results

There were no significant differences between groups at baseline (Table 1) and no differences were observed for any variable when baseline data were compared to PRE data, with the exception of a significant time effect for an increase in flexibility and vertical jump (*p* = 0.005 and *p* = 0.006, respectively). Supplement compliance was reported to be 100% as verified by collection of empty supplement containers and questioning.

### Delayed onset muscle soreness

There were no significant differences in perceived muscle soreness between groups at any time point for any of the muscle groups tested (Table 2). There were, however, significant main time effects for the rectus femoris (*p* < 0.001), vastus medialis (*p* = 0.002), vastus lateralis, (*p* = 0.001), biceps femoris (*p* = 0.007), and gluteus maximus (*p* = 0.014). Perceived muscle soreness was reported to be significantly (*p* < 0.05) greater than PRE in the rectus femoris (24, 48, and 72 hr), vastus medialis (POST, 24 hr), vastus lateralis (POST, 24, 48, and 72 hr), biceps femoris (POST, 24, 48, and 72 hr, and the gluteus maximus (POST, 24, 48 hr) of the right leg in both groups. The mean of all muscle soreness was reported to be significantly greater (*p* < 0.05) than PRE at all time-points (POST, 24, 48, and 72 hr), with no difference between groups.

**Table 2:** **Perceived muscle soreness of the right leg**

**Variable**	**Group**	**Pre DHR (day 0)**	**POST (day 0)**	**24 hr post**	**48 hr post**	**72 hr post**
Gastrocnemius	MIPS (*n* = 9)	15.7 ± 20.7	18.3 ± 19.8	17.7 ± 21.4	12.8 ± 21.1^c^	13.1 ± 17.4^c^
	CON (*n* = 10)	9.3 ± 9.7	10.6 ± 10.6	16.1 ± 13.1	11.4 ± 9.7^c^	13.0 ± 14.9^c^
Rectus Femoris*	MIPS (*n* = 9)	11.2 ± 18.5	14.7 ± 14.5	19.4 ± 15.0^abde^	14.7 ± 15.5^e^	8.5 ± 10.1^b^
	CON (*n* = 10)	10.3 ± 9.0	14.7 ± 9.6	26.1 ± 18.0^abde^	15.8 ± 14.6^e^	13.5 ± 11.1^b^
Vastus Lateralis*	MIPS (*n* = 8)	6.6 ± 10.6	16.0 ± 15.3^a^	21.8 ± 19.6^ab^	17.2 ± 17.3^a^	16.7 ± 21.2^a^
	CON (*n* = 10)	8.2 ± 5.8	14.0 ± 11.2^a^	19.9 ± 15.6^ab^	19.4 ± 15.7^a^	11.1 ± 7.9^a^
Vastus Medialis*	MIPS (*n* = 8)	11.0 ± 14.3	24.6 ± 19.7^a^	34.3 ± 24.8^abde^	20.7 ± 24.8	16.6 ± 20.9
	CON (*n* = 10)	12.6 ± 10.0	16.6 ± 12.3^a^	26.5 ± 21.0^abde^	21.4 ± 16.9	17.0 ± 13.8
Biceps Femoris*	MIPS (*n* = 9)	7.3 ± 12.8	9.45 ± 11.7^a^	21.5 ± 17.5^abe^	11.7 ± 17.8^a^	12.1 ± 14.6^a^
	CON (*n* = 10)	5.0 ± 4.0	10.2 ± 9.4^a^	19.2 ± 18.9^abe^	13.3 ± 12.0^a^	7.9 ± 9.8^a^
Gluteus Maximus*	MIPS (*n* = 9)	6.5 ± 10.2	13.6 ± 16.9^a^	20.2 ± 21.3^a^	13.2 ± 19.3^a^	8.5 ± 14.5
	CON (*n* = 10)	6.3 ± 6.5	9.0 ± 8.6^a^	10.8 ± 11.7^a^	6.6 ± 6.7^a^	8.2 ± 7.7
Mean Muscle Soreness*	MIPS (*n* = 7)	66.3 ± 88.1	110.9 ± 97.1^a^	153.3 ± 104.4^abde^	105.9 ± 106.6^a^	88.6 ± 100.6
	CON (n = 8)	55.3 ± 40.3	74.1 ± 58.6^a^	114.6 ± 90.5^abde^	86.6 ± 73.3^a^	74.4 ± 63.2

Values are expressed as mean ± SD. MIPS: Multi-Ingredient Performance Supplement; CON: Control Carbohydrate Placebo.

*p < 0.05, significant time effect.

^a^ *p* < 0.05, significantly different from Pre.

^b^ *p* < 0.05, significantly from POST.

^c^ *p* < 0.05, significantly different from 24 hr.

^d^ *p* < 0.05, significantly from 48 hr.

^e^ *p* < 0.05, significantly different from 72 hr.

### Performance measurements

There was no significant difference between maximal isometric voluntary contraction strength, maximal isokinetic strength, vertical jump, and ROM, and between baseline and PRE for MIPS and CON (data not shown). However, there was a significant time effect for an increase in ROM (*p* = 0.005) and vertical jump (*p* = 0.006) from baseline to PRE in both groups.

There were no significant group × time interactions for the MIPS and the CON across time for any of the performance measures (Table 3). There were, however, significant time effects for maximal voluntary isometric contraction for extension (*p* = 0.001) and flexion (*p* = 0.013) strength, maximal isokinetic flexion strength (*p* = 0.001), flexibility (*p* = 0.025), and vertical jump height (*p* < 0.001).

**Table 3:** **Performance measurements (** ***n*** **= 10 for MIPS and CON)**

**Variable**	**Group**	**Pre DHR (day 0)**	**POST (day 0)**	**24 hr post**	**48 hr post**	**72 hr post**
MVC Extension (60°) (N · m)*	MIPS	207 ± 52	185 ± 40	199 ± 58^b^	210 ± 49^b^	217 ± 37^bcd^
	CON	196 ± 58	184 ± 50	200 ± 58^b^	205 ± 59^b^	221 ± 64^bcd^
MVC Flexion (60°) (N · m)*	MIPS	116 ± 36^b^	107 ± 31	109 ± 33^b^	117 ± 34^b^	118 ± 26^bc^
	CON	109 ± 30^b^	99 ± 27	108 ± 30^b^	108 ± 28^b^	113 ± 33^bc^
Maximal Isokinetic Extension (180°/sec) (N · m)*	MIPS	149 ± 22^c^	149 ± 23	140 ± 20^a^	151 ± 19^c^	152 ± 18^c^
	CON	143 ± 27^c^	139 ± 18	136 ± 26^a^	140 ± 28^c^	145 ± 32^c^
Maximal Isokinetic Flexion (180°/sec) (N · m)*	MIPS	78 ± 17	77 ± 19	78 ± 16	81 ± 17^ab^	81 ± 18^ab^
	CON	70 ± 22	72 ± 19	79 ± 17	80 ± 20^ab^	82 ± 23^ab^
ROM (cm)*	MIPS	25.1 ± 11.7^c^	25.8 ± 11.3^c^	23.6 ± 12.6^a^	25.7 ± 11.4^c^	25.5 ± 11.3^c^
	CON	20.3 ± 10.6^c^	21.2 ± 10.4^c^	19.6 ± 10.5	19.3 ± 10.1	20.1 ± 10.4^c^
Vertical Jump (cm)*	MIPS	51.3 ± 7.3^c^	52.3 ± 6.6^c^	50.0 ± 18.0	50.0 ± 6.6^bc^	51.0 ± 7.1^cd^
	CON	50.0 ± 14.7^c^	50.2 ± 14.2^c^	47.4 ± 13.4	49.0 ± 13.9^bc^	50.0 ± 13.7^cd^

Values are expressed as mean ± SD.

MIPS: Multi-Ingredient Performance Supplement; CON: Control Carbohydrate Placebo; MVC: Maximal Voluntary Isometric Contraction; ROM: Range of Motion; DHR: Downhill Run; POST: Immediately Post-DHR.

*p < 0.05, significant time effect.

^a^ *p* < 0.05, significantly different from Pre.

^b^ *p* < 0.05, significantly from POST.

^c^ *p* < 0.05, significantly different from 24 hr.

^d^ *p* < 0.05, significantly from 48 hr.

### Maximal oxygen uptake

There was a significant group × time interaction for VO_2max_ between baseline and 1 week prior to the DHR (MIPS: 61.7 ± 5.3 to 63.3 ± 5.8 ml/kg/min; CON: 61.4 ± 4.9 to 60.4 ± 5.9 ml/kg/min). When evaluating the within group differences, there were no significant differences in VO_2max_; however, MIPS had an increase approaching significance (*p* = 0.06) while CON had a non-significant decrease. The standard error for the flow calibration of the Parvomedics metabolic cart was an average of 0.4%.

### Muscle damage markers

**Figure 2:**
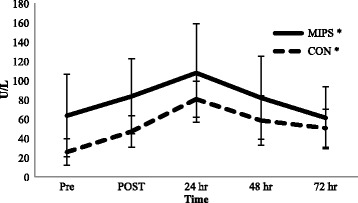
**Creatine Kinase measured in serum.** Values are expressed as mean ± SD creatine kinase levels. MIPS: Multi-Ingredient Performance Supplement; CON: Control Carbohydrate Placebo; Pre, immediately before downhill run; POST, immediately post-downhill run; 24 hr, 24 hr post-downhill run; 48 hr, 48 hr post-downhill run; 72 hr, 72 hr post-downhill run. *p < 0.05, significant time effect.

**Figure 3:**
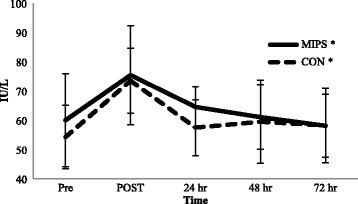
**Lactate Dehydrogenase measured in serum.** Values are expressed as mean ± SD lactate dehydrogenase levels. MIPS: Multi-Ingredient Performance Supplement; CON: Control Carbohydrate Placebo; Pre, immediately before downhill run; POST, immediately post-downhill run; 24 hr, 24 hr post-downhill run; 48 hr, 48 hr post-downhill run; 72 hr, 72 hr post-downhill run. *p < 0.05, significant time effect.

There were no significant group × time interactions for either CK or LDH from baseline to PRE (*p* = 0.056 and *p* = 0.392, respectively); however a significant time effect was observed for CK (*p* = 0.021) but not for LDH (*p* = 0.079). There was a significant time effect for both CK and LDH from PRE to 72 hr post-DHR (*p* < 0.001) (See Figures 2 and 3); however no group x time effect for CK or LDH was observed (*p* = 0.236 and *p* = 0.535, respectively). When data from both groups were collapsed, CK levels were significantly elevated from POST to 48 hr when compared to PRE values. LDH levels were significantly elevated POST before returning to PRE values at 24 hr.

## Discussion

The main findings of this study were that consumption of MIPS for 4 weeks prior to a single bout of DHR did not attenuate the changes of ratings of perceived soreness, isokinetic or isometric strength, jump performance, or ROM greater than an isocaloric placebo (CON) in highly trained male runners for up to 72 hr post-exercise. Therefore, we reject our hypothesis that the MIPS would decrease ratings of perceived soreness, improve biochemical markers of muscle damage (i.e. CK and LDH), improve muscle performance of isokinetic and isometric strength, range of motion (ROM), and jump performance greater than an isocaloric placebo in endurance-trained male runners for up to 72 hr post-DHR.

### Delayed onset muscle soreness

Delayed onset muscle soreness (DOMS) generally develops between 24-48 hr after exercise, peaks at 24-72 hr, and subsides 5-7 days post-exercise [20]. Two days after DHR, tenderness, measured by a pressure transducer, was greatest in the gluteus maximus, rectus femoris, vastus medialis, vastus lateralis, tibialis anterior, gastrocnemius and biceps femoris [21]. Our findings confirmed that the greatest amount of perceived soreness was reported during 24 and 48 hr following the DHR with no differences between groups. Therefore, it appears the selected intensity of running downhill at -5% at 75% VO_2max_ was a sufficient enough stimulus to elicit muscle soreness as shown in other studies [6,22]. In an attempt to reduce DOMS and stimulate protein synthesis following strenuous exercise supplementation with protein [22], BCAAs [9,23-25], leucine [2], and creatine [11,26] have been used. Interestingly, acute ingestion of protein (100 g of protein containing 40 g essential amino acids) immediately after a single 30 min bout of DHR had no effect on DOMS during 72 hr of recovery [22]; however supplementation with BCAAs (2.5 g of BCAAs taken both immediately prior to- and during exercise) significantly decreased DOMS from 24 to 72 hr post-endurance exercise [9]. In the present study, no significant difference in perceived soreness was reported between groups, which agrees with others using protein [22], BCAAs [10], leucine individually [2], or creatine [11,26]. The discrepancies could be due to higher individual dosages of each individual supplement in previous studies compared to our MIPS.

### Biochemical markers of muscle damage

Our findings of elevated muscle damage markers (CK and LDH) following eccentrically-based exercise support previous research [3,27-29]. Although large variability exists in the response of these muscle proteins to eccentric exercise [30-32], the concurrent elevations in subsequent pain experienced (DOMS ratings) by participants suggest the two are closely linked. Indeed, our findings support this relationship as both mean perceived muscle soreness (Table 2) and CK levels (Figure 2) were significantly elevated POST, 24, and 48 hr post-DHR in comparison to PRE. In addition, LDH levels (Figure 3) were significantly elevated POST and had a tendency to remain elevated up to 48 hr post-DHR. While the DHR protocol induced muscle soreness and damage a MIPS did not attenuate this response. Our findings agree with others examining the eccentrically-induced CK response to whey protein [5,8,22], BCAAs [10,33], leucine alone [2], caffeine [14], and creatine [11,12], but not others showing a reduction in CK as a result of creatine [12,34] or BCAA supplementation [9,24,35]. Furthermore, our findings corroborate the increased LDH response to whey protein (1.5 g/kg/day of whey protein isolate for 14 days) [5] and caffeine (4.5 mg/kg immediately prior to exercise) [14], but not the reduction of LDH as a result of BCAA supplementation (12 g/day of BCAAs for 14 days and an additional 20 g immediately prior to- and post-exercise) [35]. Again, this is likely due to different dosing of the individual ingredients within the propriety blend of our MIPS and that of other studies as well as differences in supplement timing and training status of the participants in each study.

### Performance measurements and range of motion

Decrements in performance measurements of strength (isometric and isokinetic strength) [2,5,6,23], jump height [2], and ROM [11,24,26] following eccentric exercise have been well documented. Across groups, maximal isometric strength was significantly lower at 24, 48, and 72 hr compared to immediately post-DHR. Similarly, isokinetic extension strength decreased compared to baseline at 24 hr post-DHR, returning back to baseline values at 48 hr. Interestingly, isokinetic flexion strength actually improved significantly in both groups at 48 and 72 hr post-DHR compared to baseline, suggesting a possibility of familiarization with the equipment, and/or the absence of any structural damage to the muscle fibers allowing strength to be maintained and even improve throughout the recovery period.

Etheridge et al. reported that participants supplementing with whey protein (100 g of protein containing 40 g essential amino acids) immediately after a single 30 min bout of DHR had significantly greater isometric strength at 48 hr compared to a carbohydrate supplement [22]. Controversy still exists as to whether BCAAs are effective in attenuating strength loss following EIMD. Some studies have shown that BCAAs are only effective in augmenting strength at 48 hr [9,25] after EIMD and only during the flexion phase of the contraction [9] versus placebo. Creatine also shows conflicting results for improving strength performance following EIMD. Despite our findings and others that report no change in 1-repetition maximum after EIMD with creatine use [11,26], Cooke et al. have shown that maximal isometric extension strength of the lower leg from 24 to 96 hr and isokinetic extension strength at 48 hr was significantly greater in the creatine supplementation group (0.3 g/kg/day) compared to that of a carbohydrate placebo [34]. The primary differences between studies appears to be differences in training status as the present study and others [11,26] have shown no effect of creatine supplementation with trained males, while Cooke et al. have shown improvements in strength following EIMD with untrained males [34]. Acute ingestion of protein prior to a single bout of DHR resulted in a [22] maximal voluntary isometric contraction strength that returned to normal more rapidly at 48 hr than that of placebo. Our results do not support this conclusion with the consumption of MIPS. Our findings agree with others showing that BCAA consumption (7.3 g for two days) resulted in no difference in maximal voluntary isometric contraction strength during recovery from exercise [10]. However, the aforementioned study by Etheridge et al. documented improvements in strength and power; though the authors based the intensity of the DHR on age predicted maximal heart rate (220 - age) and used recreationally active males [22].

Jump height has been found to significantly decrease at 24 and 48 hr following EIMD and remain below pre-exercise values for up to 96 hr [2,23]. Similarly, our data show decreased jump height at 24 hr following the DHR compared to all other time points with a return back to baseline values at 72 hr post-exercise, regardless of group. Eccentric exercises have been shown to significantly decrease maximal isometric contraction strength from 24 up to 120 hr compared to baseline values [5,6,9,10,22,23]. These decrements in strength from eccentric exercise are associated with a decreased ROM immediately following [11,26] and up to 96 hr [24] after exercise. ROM was slightly elevated immediately following exercise in the present study, as documented by others [12], but was significantly lower at 24 hr compared to before the DHR and was back to baseline values at 72 hr (Table 3).

As previously mentioned, decreased ROM and strength [11,26] are expected following EIMD. Our results also found a decrease in ROM at 24 hr post-DHR with ROM returning back to baseline values at 72 hr, with no differences between groups. Similarly to the findings of the present study, Nosaka et al. and Rawson et al. demonstrated no improvement in ROM between supplementation or placebo when consuming BCAAs [24] or creatine [11,26].

One interesting finding from the present study was the group x time interaction in VO_2max_ over the 4-week pre-loading period. The supplement group had an increase in VO_2max_ approaching significance at *p* = 0.064 and the CON group had a non-significant decrease in VO_2max_. The 1.6 ml/kg/min increase likely does not represent a true physiological improvement. These findings should be examined further in future research. Participants were asked not to change their training volume over the 4-week supplementation period. While training logs were not completed, verbal verification was obtained to confirm that the participants did not alter their training regimen. It is plausible that MIPS improved training quality over the 4 weeks and that this was responsible for adaptations that improved aerobic capacity. Therefore, MIPS may not improve performance in recovery but rather impacts other indices of fitness in athletes. However, this is all speculative.

Finally, it is important to note that the indirect markers of muscle damage that were used in the present study may have been limited in terms of their sensitivity, and bias, to identify specific responses to the downhill run and the effects of MIPS. This statement relates to a recent alternative perspective on changes in skeletal muscle in response to unaccustomed eccentric exercise in humans [36-38]. Specifically, these authors argue that the changes following voluntary eccentric exercise do not represent muscle damage, muscle necrosis or inflammation in human skeletal muscle but rather provide evidence for myofibrillar remodeling and adaptation [36-38]. Further, the use of indirect measures and direct antibody visualization of EIMD in previous studies, provides limited and biased information in human eccentric exercise models such as downhill running [36]. Rather, Malm and Yu [36] suggest that researchers should use proteomics to provide a powerful and unbiased protein profiling method for studying skeletal muscle adaptation to eccentric exercise. This is an important methodological issue to consider for future studies.

## Conclusions

Our results are consistent with others reporting increases in perceived soreness, CK, LDH and decreases in strength, vertical jump and ROM 24 hr after strenuous DHR exercise. However, the use of a proprietary blend MIPS had no effect on markers of muscle damage, soreness or performance compared to an isocaloric placebo. Despite the supporting body of research documenting results in the attenuation of markers of muscle damage, soreness and improved performance measurements with supplementation, other studies using protein, BCAAs, creatine, and leucine reported similar results to ours. The primary differences between our studies and those with contradicting results appear to be greater individual supplementation dosages than the proprietary blend of individual supplements in the MIPS. In addition, we used trained male endurance runners compared to that of recreationally active or untrained males in similar studies.
